# A qualitative assessment of limits of active flight in low density atmospheres

**DOI:** 10.1038/s41598-024-64114-4

**Published:** 2024-06-15

**Authors:** Mihkel Pajusalu, Sara Seager, Jingcheng Huang, Janusz J. Petkowski

**Affiliations:** 1 Department of Earth, Planetary, and Atmospheric Sciences, Massachusetts Institute of Technology, 77 Massachusetts Avenue, Cambridge, MA 02139 USA; 2 Tartu Observatory, University of Tartu, 61602 Tõravere, Estonia; 3 Department of Physics, Massachusetts Institute of Technology, 77 Massachusetts Avenue, Cambridge, MA 02139 USA; 4 Department of Aeronautics and Astronautics, Massachusetts Institute of Technology, 77 Massachusetts Avenue, Cambridge, MA 02139 USA; 5 JJ Scientific, 02-792 Warsaw, Mazowieckie Poland; 6 Faculty of Environmental Engineering, Wroclaw University of Science and Technology, 50-370 Wroclaw, Poland

**Keywords:** Exoplanets, Astrobiology

## Abstract

Exoplanet atmospheres are expected to vary significantly in thickness and chemical composition, leading to a continuum of differences in surface pressure and atmospheric density. This variability is exemplified within our Solar System, where the four rocky planets exhibit surface pressures ranging from 1 nPa on Mercury to 9.2 MPa on Venus. The direct effects and potential challenges of atmospheric pressure and density on life have rarely been discussed. For instance, atmospheric density directly affects the possibility of active flight in organisms, a critical factor since without it, dispersing across extensive and inhospitable terrains becomes a major limitation for the expansion of complex life. In this paper, we propose the existence of a critical atmospheric density threshold below which active flight is unfeasible, significantly impacting biosphere development. To qualitatively assess this threshold and differentiate it from energy availability constraints, we analyze the limits of active flight on Earth, using the common fruit fly, *Drosophila melanogaster*, as a model organism. We subjected *Drosophila melanogaster* to various atmospheric density scenarios and reviewed previous data on flight limitations. Our observations show that flies in an N_2_-enriched environment recover active flying abilities more efficiently than those in a helium-enriched environment, highlighting behavioral differences attributable to atmospheric density vs. oxygen deprivation.

## Introduction

Active flight is an energetically costly endeavor that requires the evolution of tissues that are capable of high energetic output—an example of such a tissue are muscles of animals. For muscles (or physiologically analogous tissues) to evolve, complex multicellular life has to arise first. We explore the limits of active flight of the common fruit fly *Drosophila melanogaster* as an example tool to study limits of active flight in low density atmosphere environments. We discuss if, and how, low density atmospheres put hard constraints on the evolution of active flight on planets with low density atmospheres.

We start with the introduction, by briefly describing critical issues and evolutionary inventions that are needed for active flight to evolve in a biosphere: (1) the possibility of the emergence of the complex multicellular life, with a tissue capable of generating significant energy output (i.e. muscles, or muscle analogs) (Section "Introduction to complex life") and (2) the possibility of the universality of the active flight evolution by complex life, in any exoplanet scenarios (Section "Active flight as an inevitable attribute of complex life?"), followed by the review of ecological importance of the active flight for the entire biosphere, including non-flying organisms.

We follow the introduction with the description of our experiments on the common fruit fly *Drosophila melanogaster* (Section "The experimental setup and operation"), where we determine the atmospheric density limit for the flies and show that the limits of active flight for *D. melanogaster* is a direct effect of the atmospheric density, not the availability of O_2_. We rule out the influence of O_2_ availability by conducting experiments that vary the gas composition in the environment so that we can control atmospheric density and O_2_ availability separately (Section "Observed fruit fly behavior"). We conclude the paper with a discussion of the broader context of powered flight and complex life in exoplanet atmospheres (Section “Summary and Conclusions”).

### Introduction to complex life

Complex life is often defined as obligatorily multicellular^1–3^. As a consequence of its multicellularity complex life is generally larger than single-cellular life and has a distinctive developmental program. To thrive, multicellular organisms require a diverse ecosystem with multiple trophic levels that occupy many diverse ecological niches. On Earth, complex multicellular life are animals, plants and multicellular fungi. All complex multicellular organisms on Earth belong to one domain of life: *Eukarya*. Chemistry and the overall cell structure are less diagnostic of eukaryotes and complex multicellular animals, than one would at first assume. Eukaryotic cell chemical composition and cell structure do not preclude if a cell can build a complex multicellular organism or not. In general, prokaryotes (i.e. bacteria and archaea) are at least as chemically diverse and adaptable as eukaryotes^4^ and there are no apparent reasons why prokaryotic cells could not build a complex multicellular organisms. In fact, prokaryotes have developed rudimentary forms of multicellular complexity^5–9^. Once life originates on a planet, many key evolutionary innovations such as the transition from single-cellular to multicellular life are likely to occur, as there are many evolutionary routes leading from simple single-celled organisms to complex multicellular organisms (provided that the planet retains habitable conditions for a suitably long time)^1,2^.

However, active flight that multicellular life exhibits is an energetically costly endeavor and the limitations of bioenergetics, and specifically the reliance on oxygen (O_2_), could play the dominant role in the propensity of complex multicellular life on exoplanets. Indeed, Eukaryotes appear to be able to generate significantly more energy per gene than prokaryotes^4^. Energetics however is not the only potential barrier to the emergence of complex life, the type of genetic control and genome complexity employed by Eukaryotes might be equally, if not more, important^4,10^. We discuss the bioenergetic limitations of complex multicellular life, with the special emphasis on the role of O_2_, briefly below.

Is O_2_ absolutely required for complex multicellular life? Some scientists argue that complex life without O_2_ is impossible, and that significant amounts of O_2_ in the air and ocean are essential for the evolution of multicellular organisms, and that this need is universal^11,12^. They argue that anaerobic metabolism is too inefficient^11^. Indeed, the generally low energy output from anaerobic respiration is a limitation. However alternative energetic strategies are potentially possible.

For example, if a complex multicellular organism collects, stores and concentrates critical energy metabolites then its high energy needs could be easily met^13^. One such possible, although highly speculative, metabolic strategy relies on collecting, storing and concentrating sulfates (and venting CO_2_ and H_2_S)^14^. Even in Earth’s O_2_-rich environment, there are many examples of multicellular organisms that can live in concentrations of O_2_ as low as just few percent or less (e.g. summarized in^1^), or can adapt to very low O_2_ conditions as a result of directed evolution experiments (e.g. even very active animals like fruit flies, *D. melanogaster*, can thrive and effectively fly and breed in O_2_ concentration as low as ~ 4%^15^), with some parasitic multicellular organisms not requiring O_2_ at all^16^. It is therefore in principle possible that the nearly absolute reliance on O_2_ that we see in complex multicellular organisms here on Earth is a result of an adaptation of complex multicellular organisms to dominant and ubiquitous O_2_-rich environment of Earth and not an absolute, inherent, universal limitation of complex multicellular life.

### Active flight as an inevitable attribute of complex life?

Active flight (also called powered flight) is a type of animal flight that uses muscles to generate aerodynamic force that is sufficient to generate enough lift and thrust. The ability of active flight allows the animal to ascend without the support of the rising air (in contrast to unpowered flight like gliding), and in that regard active flight is much more energetically costly than any other type of flight.

The recurrent evolution of active flight among different animals on Earth is a textbook example of convergent evolution.

Convergent evolution is an independent evolution of analogous features, morphological forms or biological functions, which appear in unrelated species that were not present in the last common ancestor of those species. Note that often those unrelated species are not only separated from each other in space but also in time and often belong to different eras in geological history of the planet.

On Earth, only animals (which have highly metabolically active muscle tissues) evolved the ability of active flight, although, as we discuss below, many branches of the animal kingdom did so completely independently from each another. Therefore, the question whether the evolution of active flight is a rare event, or whether the emergence of active flight is an evolutionary inevitability that is likely to happen on any exoplanet that allows for the formation of complex multicellular life is valid. Before we attempt an answer to this question, we briefly discuss the evolutionary span of the active flight among animals on Earth and the evolutionary advantages and ecological importance of its development.

Active flight has evolved several times among animals. First in insects (approx. 400 Mya^17^) and reptiles (pterosaurs—230 Mya^18^), and subsequently in birds (150 Mya^19^), and most recently in mammals (bats—probably around 60 Mya (e.g.^20–22^)).

Insects (*Insecta*) are the first animals to evolve active flight and also the only invertebrates that have evolved the capability of active flight. Within the *Insecta* class the ability of active flight has single origin in the clade *Pterygota* that contains all flying insects distributed into two large sister groups (*Paleoptera* and *Neoptera*)^23,24^. Insects clearly are the most numerous group of animals to utilize active flight, numbering in millions of flight-capable species.

The reptile pterosaurs were the first actively flying vertebrates, which likely were capable of very active and sophisticated flying techniques, likely from a very early age^25^. There were hundreds of species of pterosaurs most of which were intermittent wing flappers and soarers^18^. The largest flying animals to ever exist on Earth are pterosaurs, e.g. *Quetzalcoatlus northropi*, with a wingspan of ~ 12 m and total weight of ~ 225 kg^26^. Another extinct set of reptiles that likely were capable of active flight were theropods. Theropods are not birds, although they are closely related to them. Theropods evolved active flight independently across different lineages^27^.

There are approximately 18,000 species of birds on Earth^28^. The great majority use active flight (including soaring), and only a few dozen species have lost the ability to fly^29^. Birds are by far the most sophisticated active fliers in the entire vertebrate (*Vertebrata*) subphylum.

Actively flying mammals are rare and are only represented by bats. There are approximately 1,400 bat species, which corresponds to approx. 20% of all known mammalian species^30,31^.

At first glance the fact that convergent evolution of active flight in animals arose only several times suggests that the ability to fly is difficult to evolve. Flight evolution happened on a wide scale only four times during the last 350 Myr, and only among members of one kingdom (*Animalia*) of multicellular organisms.

However, active flight has unique evolutionary and ecological advantages for any complex multicellular organism that pursues it. Those advantages could offset the high energetic cost of development and maintenance of expensive muscle tissues required for unassisted flying. After all, although active flying is energetically costly, when we consider the distance flying organisms can cover per unit of time, the overall cost per distance traveled is quite minimal. We briefly discuss such ecological and evolutionary advantages next.

Active flight is one of the best strategies to rapidly evade predators and remain out of their reach for long periods of time. It allows for migration over very large geographical distances, often into areas that are not easily accessible for non-flying animals. Active flight grants better access to new feeding grounds and nesting sites. Finally, active flight allows to avoid natural disasters. For example, one could speculate that it is due to the ability to fly that the only “dinosaurs” that survived the K/T (Cretaceous–Tertiary) extinction 66 Mya are birds, simply because they were able to fly away to the less affected areas.

Flying animals have also a global beneficial or even crucial role for the whole ecosystem of the inhabited planet. Examples of such beneficial effect include: widespread plant pollination, transfer of plant seeds, fungal spores over long distances and even transfer of fish eggs between different bodies of water, and many other examples.

The evolutionary advantages and ecological importance of the development of active flight by animals on Earth suggests that its discovery could be, to an extent, universal to the majority, if not all, exoplanet environments that allow for the formation of complex life.

One could ask a question if there are any planetary ecosystems that would prevent development of active flight, or make it significantly less likely.

The first such environment could be water worlds (planets with a global ocean and no dry land). It is important to realize however that both water in an ocean and air in the atmosphere are governed by fluid dynamics. Though at the first glance counterintuitive, they both share quite a few similarities in terms of their overall functional ecology. A good example of such ecological similarity is a predatory strategy of hawking, a feeding strategy that, e.g., involves catching flying insects in the mid-air during flight. Hawking is a strategy that is analogous to chasing prey in open pelagic waters that is utilized by many marine animals. Based on such similarities, we could speculate that applying similar physical principles between the two media to build a functional wing for active flight is possible, even on a planet completely covered in ocean.

This rather bold statement is supported by limited data on the capability of active flight of fish. Due to the obvious apparent limitations of the water environment, fish are often overlooked in the assessment of active flight capabilities. There are however, reports that several species of freshwater hatchetfish (*Gasteropelecidae*) are capable of jumping out of the water and limited active flight for short distances. They can propel themselves through the air for up to 6 m by actively flapping their pectoral fins^32^ (note that the marine fish species, commonly referred to as “flying fish” (*Exocoetidae*), are not actively flying and are only capable of unpowered, gliding flight). Hatchetfish active flight is possible due to the uniquely developed sternal region, pectoral fins and extremely powerful associated pectoral fin muscles that account for approx. one-quarter of the total body weight of the average hatchetfish.

The second potential factor hindering the ability of flight could be surface gravity on the planet, as higher surface gravity would require proportionally more thrust to be generated. At the same time, however, higher gravity would increase the surface pressure and thus atmospheric density, which could counteract this challenge.

Finally, the third possible environment that could limit the development of the active flight are planets with low density atmospheres. Those planets might create a special difficulty for active fliers, as it might be difficult for them to generate sufficient lift and thrust needed to ascend, that generally is not a limitation on planets with dense atmospheres.

### Motivation for studying atmospheric density as the main variable for the existence of active flight

Based on the variation of conditions supporting flight on Earth, the main limitation seems to be the atmospheric density. As all forms of powered flight require thrust to be generated and for covering significant distances this thrust has to come from changing the velocity of air around the flying organism. Moreover, the lower the atmospheric density, the larger volume of air that needs to be moved or it would need to be moved at a larger velocity.

As can be seen on Earth, long duration flight also requires lift to be generated, and successful lift generation allows birds to achieve very long flight paths. For shorter duration, however, lift is not a requirement and insects can fly mainly by generating vectorized thrust that has to overcome the force of their weight.

We propose that active flight is possible if there is a sufficient atmospheric density present (in an atmosphere suitable for life). Here we focus on the mechanisms of generating thrust. Based on the data of active flight on Earth, generating thrust will be a much more significant challenge than generating lift, because the highest-flying organisms are birds, which utilize lift, while the flight ceiling of insects, which utilize thrust, seems to be limited by the amount of thrust that can be generated. The flight ceiling limitations of insects are supported by the observation that often losing the ability to fly is one of the first adaptations of insects to the high altitude habitats^33^ (with a notable exception of alpine bumblebee *Bombus impetuosus*^34^).

We are therefore prompted to ask: *“What are the limits of active flight in low density atmospheres?”.*

For this, we conducted a set of experiments with *D. melanogaster* to investigate the qualitative limits of active flight due to atmospheric density and to distinguish these effects from problems that stem from energy availability (low O_2_ environment).

## Results and discussion

### The experimental setup and operation

**Figure 1:**
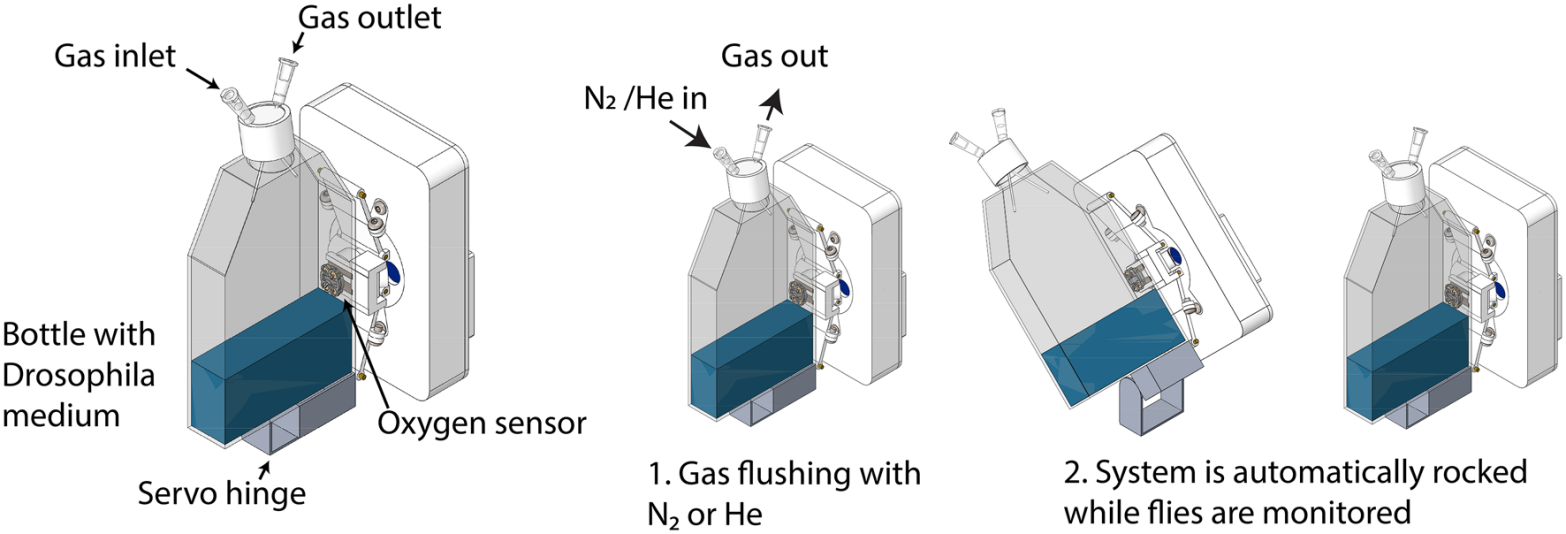
Schematic of the experimental setup.

To ascertain the limits of active flight, we developed a setup designed to observe the flight of common fruit fly *D. melanogaster* in a variety of atmospheric compositions at ambient pressure (Fig. 1). The experiment was conducted in a small container, allowing for a rapid change of the atmosphere to yield a homogeneous gas mixture. This approach also allows for the experiment to be readily reproduced, as we used standard laboratory equipment and supplies.

We conducted two types of experiments. The first set of experiments was to observe flight of *D. melanogaster* in conditions where a fraction of the air within the container was replaced with N_2_ to test the limitations to flight due to oxygen (O_2_) deprivation, with a marginal change in atmospheric density (due to the difference between the density of N_2_ and average density of air being small). The second experiment was to probe the limitations and effects on the *D. melanogaster* flight that come from low atmospheric density (a fraction of atmosphere replaced by helium), while using the first experiment’s results to distinguish these effects from the effects of reduced O_2_ availability. Both types of experiments started with a culture of fruit flies with their surroundings flushed with room air to demonstrate high activity and flight capability in those conditions. To promote flight, the setup was shaken on a continuous cycle (Fig. 1; see also Figs. 6 and 7 in the Materials and Methods section).

**Figure 2:**
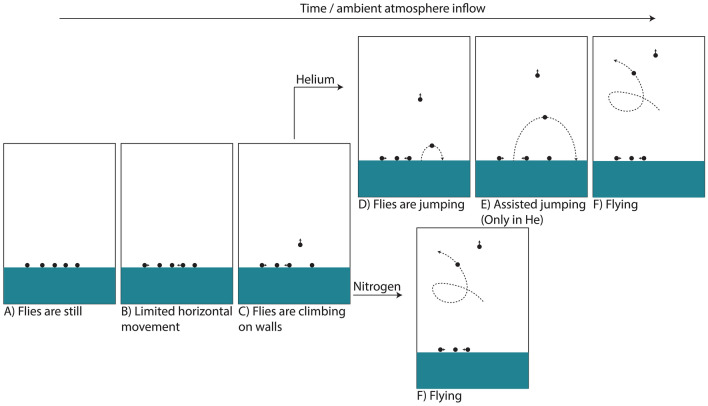
Types of fly behavior observed in the experiment. At the lowest O_2_ concentrations, i.e. after flushing the experimental bottle with N_2_ or He, flies are immobile (**A**). When the O_2_ concentration rises, flies start to move horizontally (**B**) and eventually climb on the container walls (**C**). At a combination of conditions, flies can start to jump (**D**) and use their wings to jump higher, to glide, or to briefly hover (**E**). When atmospheric density is high enough (and enough O_2_ is available) flies start to fly (**F**), i.e. we observe trajectories that can significantly deviate from a parabolic trajectory. Note that the stages D and E were only observed in He-containing experiments; flies in the N_2_-containing experiments transitioned directly from climbing on the container walls (**C**) to flying (**F**).

**Figure 3:**
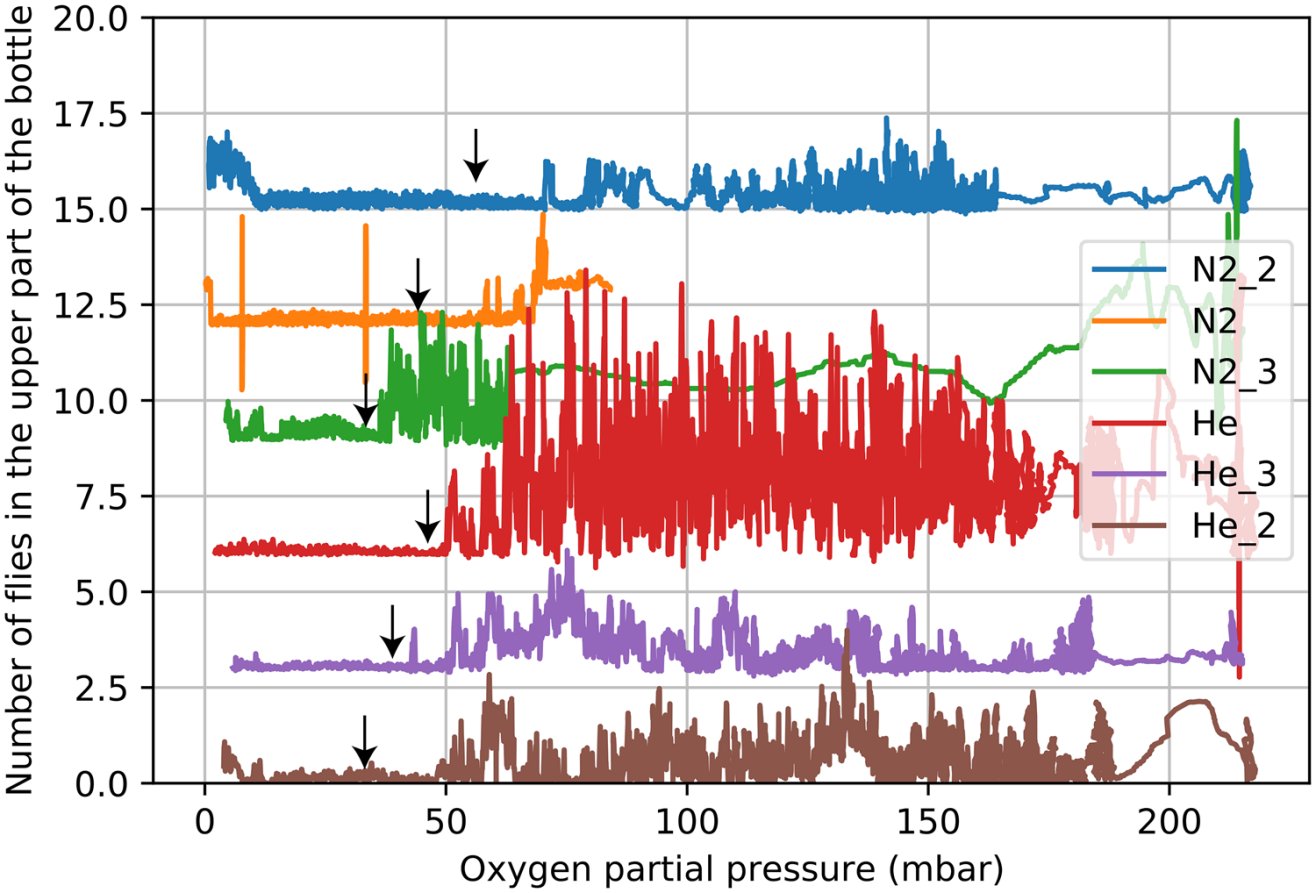
Fly activity determined automatically via machine learning (y-axis) as a function of oxygen partial pressure (x-axis) in all monitored experiments with N_2_ and He (number of identified flies climbing on the walls of the vessel) vs O_2_ partial pressure. The first recorded walking movement of flies is marked by black arrows. Plots have been shifted vertically by multiples of 2.6 for better visual separation. In all experiments, the number of active flies drops to near zero when the chamber is flushed with N_2_ or He, producing a flat-line area in the graph. Once air diffuses back in, flies become active and they start to climb on the walls of the container, producing the higher signal. Flies become active between 30 and 50 mbar of O_2_, in both He and N_2_, showing that the flight behavior reported on Fig. 4 is decoupled from availability of O_2._ Helium experiments are much more consistent than the N_2_ experiments, perhaps because helium diffuses more uniformly.

In the first phase of a given experiment, the container was flushed with the gas under study (N_2_ or He). In the second phase of an experiment, that is after flushing, we allowed ambient air to diffuse back into the bottle at a low rate. This allowed us to observe flies’ flight behavior during their recovery from the less-dense and O_2_-deprived atmosphere as the atmospheric conditions approached ambient air (a continuum of compositions, i.e., from a nearly pure He or N_2_ atmosphere to ambient composition of approximately 78% N_2_, 21% O_2_ and 1% Ar).

### Observed fruit fly behavior

During the experiments, we observed several different types of behavior, which we can link to changes in O_2_ concentration and atmospheric density (Fig. 2). During the experiment, we only monitored the O_2_ concentration in the container and used it as a proxy for determining concentrations of other gases and thus also the atmospheric density in the experiment. Due to the qualitative nature of the experiment we did not take water vapor saturation into account, which can also change the overall air density.

**Figure 4:**
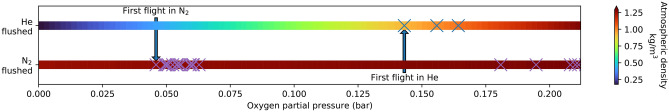
Active flight events as a function of oxygen partial pressure. The color of the bar in the background indicates the density of the gas mixture in the experimental bottle, the x-axis indicates oxygen availability for metabolism as partial pressure (bar). The crosses (X) show active flight events at a given oxygen partial pressure in experiments originally flushed with N_2_ (purple X) and He (blue X) during ambient atmosphere inflow. First active flight in the low density atmosphere (containing He) happens much later, at a significantly higher oxygen availability than in N_2_ experiment, decoupling the effect of the oxygen availability and atmospheric density on the initiation of active flight.

To identify different fly behaviors, we developed a machine-learning based workflow, which automatically identified events where flies were in the free 3D space within the bottle (not climbing on the walls). These events were then manually classified into different types of behavior. This approach allowed us to find events and behaviors we did not notice when looking at the experiment videos directly. This is due to general difficulty of observing individual fly behavior when many flies are moving in the container at the same time. Such an approach also reduces observer bias, as detecting movements among large numbers of flies is difficult by eye.

The identified fly behavior stages are as follows (Fig. 2):

A. Very low oxygen partial pressures (nearly pure N_2_ or He): Flies are in an O_2_-deprivation shock state and completely immobilized.
B. With rising O_2_ partial pressures, flies start to sluggishly move, this is generally only on horizontal surfaces.
C. Higher O_2_ concentrations start to facilitate flies climbing on the container walls.
D. In He, higher O_2_ concentrations (exact values described below) lead to jumping behavior. This behavior was only observed in atmospheres containing helium, i.e., low density atmospheres. In the N_2_ experiments, the flies proceeded straight to active flight (stage F).
E. When flies are fully active and the atmosphere becomes more dense as the experiment progresses (see below for exact values) “assisted jumping” can be observed. This is characterized by significantly higher jumps, as compared to behavior recorded in stage D, with clearly moving wings, but the flight follows a roughly parabolic trajectory, showing that thrust is not enough to overcome the gravitational force and essentially the effect is comparable to jumping in a lower gravity environment (thrust can be subtracted from the gravity). This behavior was only observed in atmospheres containing helium.
F. At a certain combination of O_2_ concentration and atmospheric density, thrust overcomes gravity and sustained non-parabolic fight is possible. The main point of these experiments is to find these combinations of O_2_ concentration and atmospheric density.

To relate the observed fly behaviors with atmospheric density and to separate the effects of atmospheric density from availability of energy (in the context of aerobic metabolism this means availability of O_2_), we monitored changes in fly behavior while ambient atmosphere was diffusing into the experimental vessel.

To monitor the general state of fly activity and its relation to O_2_ concentration, we plotted the number of flies climbing on the experimental container walls, which was determined by the machine learning approach described in the methods section, against O_2_ partial pressure (Fig. 3). Note that the general activity of flies and the number of live flies varied in experiments, because we could not control the exact number of flies we transferred to the container and the number of the flies surviving the transfer. However, we note that the overall results and the conclusions are not affected by the number of flies undergoing behavioral changes. To increase the robustness of the activity limit determination, we also manually checked the dataset to identify the first clear intentional actively walking flies (movement of at least a distance of their own body length) in each dataset.

**Figure 5:**
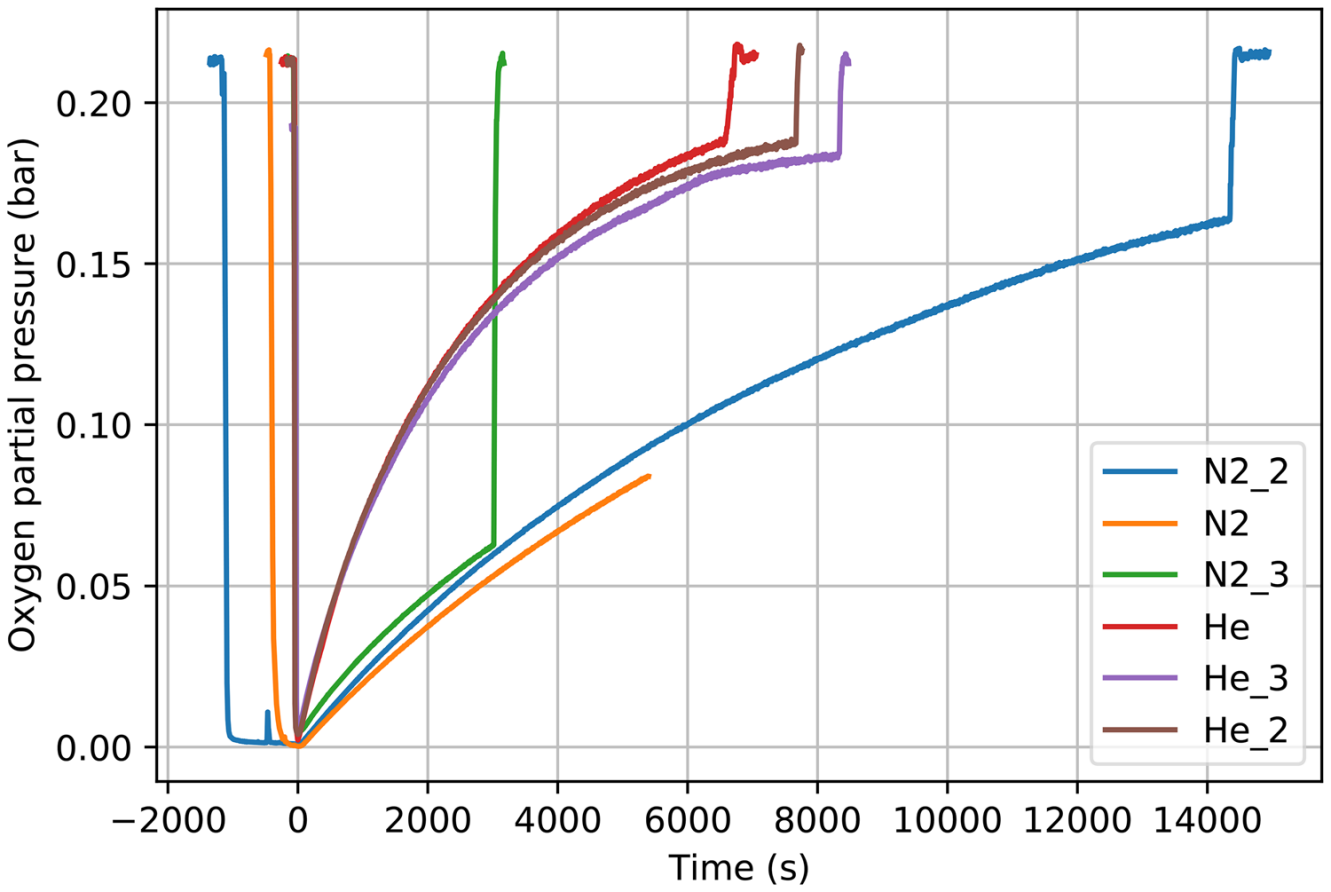
Graph of O_2_ partial pressures within the experimental bottles, showing the progression of the experiments and atmosphere replacement rates for He and N_2_ across all experiments. The rapid drop on the far-left side of the graph shows rapid and efficient flushing, i.e. removal of air (and therefore O_2_) from the bottles and substituting it with N_2_ and He respectively, the latter part shows ambient air entering back into the experimental bottle. The O_2_ partial pressures were measured with custom-made oxygen sensor^36^.

To determine the limits of flight, we used a combination of neural networks and stereo vision (see the Materials and Methods section for the exact methods). The candidate flights detected by this method have been manually evaluated and classified to distinguish flies that fall, presumably due to unsuccessful flight attempts, from flies that actually actively fly, as well as distinguishing cases where flies clearly produce thrust and therefore jump higher, briefly hover, or drop slowly.

The experimental results are summarized in Table 1. From these results we conclude that atmospheric density is the limiting factor for the ability of *D. melanogaster* to actively fly in the He experiments and not the availability of O_2_. This conclusion is supported by the following result: in the N_2_ flushed case, flight capability recovered at 46 mbar of O_2_ partial pressure (gas density in container at 1.26 kg/m^3^) and in He, lowest partial pressure for flight was 142 mbar of O_2_ partial pressure (gas density in container at 0.93 kg/m^3^) (Fig. 4; Table 1).

**Table 1:** Summary of the results of the experiment. Minimal O_2_ pressures were taken over datasets (3 datasets for He and 3 datasets for N_2_ were used).

Experiment (initial atmosphere in the experimental bottle chamber)		N_2_	He
First documented restoration of activity (one fly walking at least one body length)	O_2_ pressure (mbar)	33	33
	Density (kg/m^3^)	1.26	0.35
Climbing on walls	O_2_ pressure (mbar)	38	48
	Density (kg/m^3^)	1.26	0.43
First documented flight	O_2_ pressure (mbar)	46	142
	Density (kg/m^3^)	1.26	0.93

**Figure 6:**
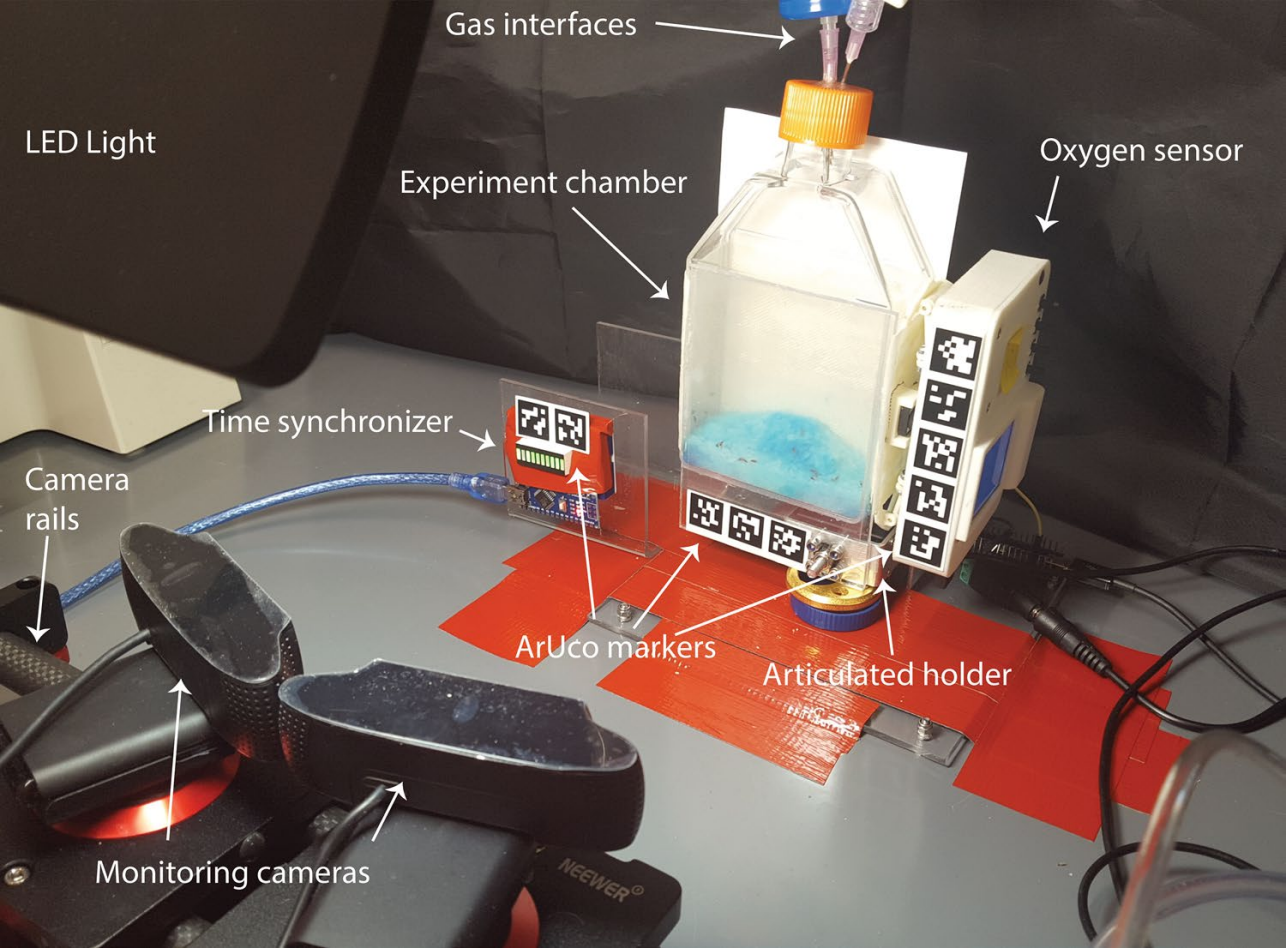
Observational setup. The flies were grown in a cell culture bottle placed vertically in a motorized holder, which formed an experimental chamber with controllable environment. The holder also allowed to attach an O_2_ sensor reliably and included an articulated stage to shake the chamber. Due to strong mechanical stresses encountered during shaking, the bottle holder had to be taped to the surface of the table. The setup was monitored using two webcams on a rail setup and tracking markers were placed on the scene for easier automatic geometry determination.

At atmospheric densities below the limit at which first flight was detected, in He flushed case, it is clear that the flies were attempting to fly, but could not generate enough lift, resulting in a behavior we call “assisted jumping” (Fig. 2). At lower atmospheric density, flies were also clearly capable of modifying their rate of falling and direction of the fall after attempted flight.

In principle changes in gaseous composition of the air could affect the fly’s nervous system and change the flight behavior.

The changes in gas mixture, that are independent of O_2_ availability, could influence the results of the presented experiments, for example by affecting the time point when *D. melanogaster* first attempts flight.

We note however that we designed our experiments to control for such an eventuality. As a control, we monitor the behavior of a large number of flies in a chemically inert N_2_ gas environment. We register clear differences in flies’ behavior between the control N_2_ conditions and the low-density He-filled experimental chamber (Figs. 3 and 4). In our experiments helium and nitrogen do not appear to cause any physiological effects to *Drosophila*’s brain performance that go beyond the temporary oxygen deprivation. We expect the oxygen deprivation effect on the fly nervous system to be similar in both helium and nitrogen experiments. This conclusion is supported by our observations. We see a clear difference between the fly behavior in nitrogen and helium experiments, which shows that oxygen deprivation itself does not cause the observed differences (Figs. 3 and 4). One can argue that nitrogen and helium could have different effects on the fly’s brain that are as of yet uncharacterized and unknown, causing different fly behavior. We note that both gases are chemically inert (in the context of this experiment), so if such effect exists, it cannot be due to reactivity of the gasses with the *D. melanogaster* brain, but rather it has to come from a physical effect unrelated to the chemistry of the tested gasses.

## Summary and conclusions

The results show that *D. melanogaster* flies need to be in an atmosphere with a density of at least 0.93 kg/m^3^ (72% of ambient air density at sea level), predicting that the flight ceiling of *D. melanogaster* is around 3 km from sea level. To compare our inferred flight ceiling of *D. melanogaster* to the flight ceiling of different animals, we assembled Table S1 in the SI. Note that birds have been found flying at ~ 12 km altitudes, showing that advanced flight of vertebrates has far fewer limitations than the flight of insects^35^. From life on Earth, we know that the minimum atmospheric density per class of animals is roughly 0.36 kg/m^3^ (as illustrated by the flight ceiling altitude of birds like *Gyps rueppellii*^35^, or insects like alpine bumblebee *Bombus impetuosus*^34^) (Table S1). While our research does not identify absolute limits for active flight in low density atmospheres, such limits do have to exist. For active flight to exist it requires a mechanism leading to the displacement of air as the flight progresses. The displacement of air on the other hand requires a specific minimal air density, putting a hard limit for air density for active flight. Our results show what such an air density limit could be. We note that life could have evolved specialized adaptations, that have no precedent on Earth, that could make active flight possible on exoplanets with low density atmospheres.

Our results have implications to exoplanets and exoplanet habitability. The low atmospheric density is expected to challenge the active flight capability of organisms, force life to develop specialized adaptations, even if sufficient amounts of energy can be generated, but likely will not prevent it entirely (Fig. 5).

## Materials and methods

**Figure 7:**
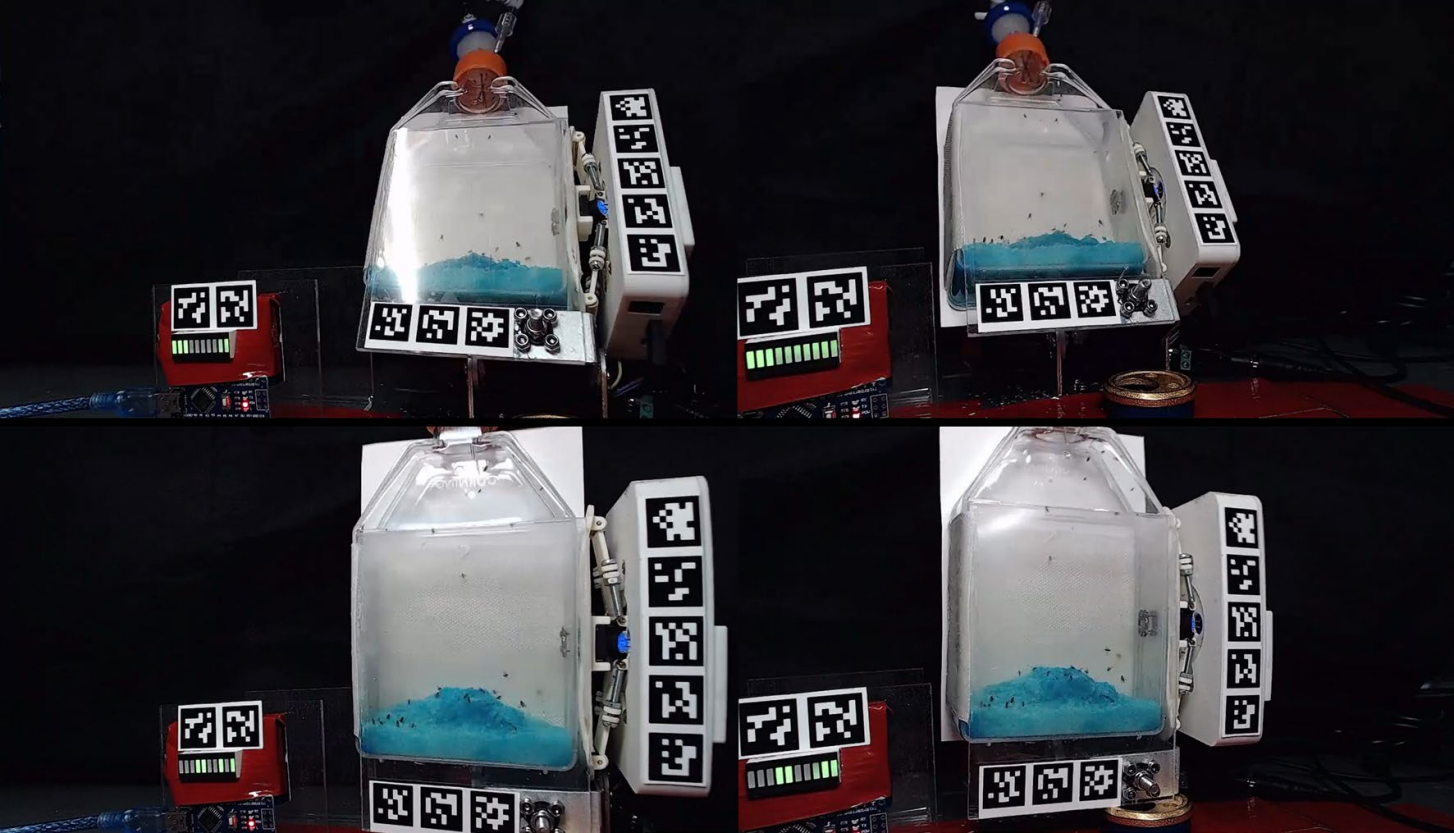
Setup in operation. The top part shows the view from the stereo cameras when the bottle is bent to the furthermost back state. The bottom part shows the setup just after reaching the fully upright position.

The experiments were conducted in a custom-built setup (see Figs. 1, 6 and 7 for reference), which allows monitoring of *D. melanogaster* flight in artificial atmospheres. The core of the setup is a cell culture bottle (75 cm^2^ rectangular canted neck cell culture flask with vented cap, product #430641 from Corning Inc., Corning NY), to which instant *D. melanogaster* medium (formula 4–24, blue, from Carolina, Burlington, NC) was placed along with deionized water and Fleischmann active dry yeast (from ACH Food Companies, Oakbrook Terrace, IL). For the experiment, we used wild-type, Oregon R strain of *D. melanogaster* (item 172100 from Carolina, Burlington, NC).

The cell culture bottle was placed in a custom-made holder, based on an aluminum fixture and a servo motor (modified Hobbypark HDR315M 15 kg Digital High Torque Robot Servo Motor with U Mounting, from HobbyPark, purchased through Amazon.com). To form a stable container, a transparent acrylic sheet (0.25" thick) was mounted to the bracket and a sheet of epoxy-impregnated fiberglass was molded around the bottle to form a tight and repeatable fit. Due to the flexibility of the fiberglass enclosure, it was possible to exchange bottles in the setup, when needed. One side of the fiberglass fixture was modified to hold a custom-made oxygen sensor (see^36^), which was modified to work with Ocean Optics FOSPOR patches (RE-FOS-8-KIT from Ocean Optics, now Ocean Insights, Largo, FL) by replacing the dye, previously manually applied to the magnetic holder, with a FOSPOR sticker, and by modifying modulation frequency of the excitation LED. This allowed oxygen partial pressure measurement from trace amounts to ambient levels, covering all conditions relevant to the experiment.

For each experiment, the bottle was flushed with either nitrogen or helium and after flushing the *D. melanogaster* behavior was monitored as oxygen was seeping back in to the bottle. Plots of oxygen partial pressure vs. time are shown in Fig. 5, showing that the experiments with N_2_ and He were separately very similar regarding to gas inflow, but He diffused out significantly faster (allowing for inflow of room air) (Fig. 5). The faster diffusion rate is not expected to change any conclusions from this experiment. Both He and N_2_ experiments were conducted in triplicate (He_1-He_3 and N2_1-N2_3 respectively). We do not expect these differences between N_2_ and He experiments to affect the overall results, as the aim of this article is to qualitatively evaluate the process, although we note that the He experiments seem to be more consistent with each other. The higher air inflow speed in case of He would be expected to promote faster flight recovery.

For monitoring, two Logitech C920 webcams (from Logitech, Lausanne, Switzerland) were used. To be able to capture the data streams from both cameras at full resolution and to store the data at maximum framerate (1080P at 30 frames per second), a custom GStreamer (https://gstreamer.freedesktop.org/ ) plugin was written, allowing to capture and save JPEG frames compressed within the camera modules straight to storage media without any, potentially detrimental and resource intensive, intermediate processing. All further image processing was performed on these JPEG frames, offline, after the conclusion of the experiment. As the cameras were not synchronized on hardware level, a custom timing synchronized, made out of a 10 segment LED strip and an Arduino Nano was used to provide a visual timestamp to every image captured. The camera synchronization system was, still, not used in later experiments, as monitoring computer clock while taking images was indistinguishable from the LED strip method in practical tests and the LED strip method was dependent on lighting conditions, failing in some cases. Therefore, final time synchronization was provided by the computer clock timestamp (Python’s time.time()).

In order to robustly determine the geometry of the system, ARUCo markers were applied to the visible surfaces^37,38^. For better visibility of the flies, a white paper sheet was placed behind the bottle.

During data processing, the O_2_ partial pressure was calculated from the O_2_ sensor readings and referenced with the stereo images. When this image dataset was compiled as a video, it allowed for preliminary visual evaluation of flight capability.

We started with a DarkNet implementation of YOLOv3-Tiny classifier^39^, which was custom trained on annotated images obtained during the experiment, but ended up using Mask-RCNN from Facebook Research (https://github.com/facebookresearch/maskrcnn-benchmark ) for the actual data analysis. We trained Mask-RCNN (with Resnet 101 backbone) by annotating images with flies manually (332 images for training and 82 for validation). The network had previously been trained on COCO (Common Objects in Context) and so training on the fly dataset converged quickly (20 training iterations were generally enough). Data was also pre-processed to only show the area where flies can be found on the image and rest of the image was masked out.

Our data pipeline was as follows:

1. Random image pairs with similar enough timestamps (within 10 ms of each other) were selected from the JPEG images captured and Mask-RCNN was run on them (6 parallel threads were used, utilizing an Nvidia RTX 3090 and an Nvidia RTX 2080 Ti on the same computer).
2. The program used the Mask-RCNN detections together with a preliminary stereo calibration (using checkerboard calibration) and locations of the ARUCo markers to solve the bundle adjustment problem and to localize the flies in 3D space. As individual flies were not matchable by the detection data alone, the algorithm matched all detected flies against all detected flies and solved the stereo reprojections. The final 3D locations of flies were determined as points which resulted in a sub-threshold reprojection error.
3. The resulting 3D positions of flies in the reference frame tied to the bottle (axes determined by the Charuco markers) were saved to hard drive for later processing. Images where the bottle was moving during the detection were rejected, because these would produce erroneous measurements due to motion blur and rolling shutter effects.
4. The 3D positions of flies were filtered: one filter was just detecting flies in the upper portion of the bottle. This produced the activity plots on Fig. 3. The second one detected flies that were not on the walls of the bottle (meaning that they had to be either flying or falling).
5. Flight detection dataset was filtered to exclude false positives by looking at rapid movement (points that seemed to be erroneously inside the bottle but were actually still on the walls remained stationary between frames, while flight always caused rapid movement).
6. The filtered flight detection dataset was then analyzed to separate different fly behaviors by eye to identify the behavior class associated with each individual event.

The temporally dynamic point cloud was then processed into individual fly tracks using Trackpy (http://soft-matter.github.io/trackpy/v0.4.2/index.html# ), giving individual flight paths of flies. In order to reduce the computational complexity of tracking, the point cloud was processed in two steps, first tracking slow-moving (crawling) flies and removing them from the point cloud and then tracking the reminder of fast-moving points separately. All other image processing tasks were performed using OpenCV 4 (https://opencv.org/).

## Data availability statement

The raw data is not currently freely available due to it large size (a few terabytes), making the raw data distribution a complex endeavor. The authors are willing to provide the original datasets on request. The original video files are available for download from Zenodo at https://zenodo.org/records/11060392.

### Supplementary Information

Supplementary Information.
