# Supplementary material for: A qualitative assessment of limits of active flight in low density atmospheres
Source: Sci Rep. 2024 Jun 15;14:13823. doi: 10.1038/s41598-024-64114-4 (PMC11180128; doi:10.1038/s41598-024-64114-4)
Supplement: Supplementary file 1 — Supplementary Information. [file 41598_2024_64114_MOESM1_ESM.docx]

Supplementary Information

**A qualitative assessment of limits of active flight in low density atmospheres**

Mihkel Pajusalu^1,2,*^, Sara Seager^1,3,4^, Jingcheng Huang^1^, and Janusz J. Petkowski^1,5,6^

^1^ Department of Earth, Planetary, and Atmospheric Sciences, Massachusetts Institute of Technology, 77 Massachusetts. Avenue., Cambridge, MA 02139, USA

^2^ Tartu Observatory, University of Tartu, 61602 Tõravere, Estonia

^3^ Department of Physics, Massachusetts Institute of Technology, 77 Massachusetts. Avenue., Cambridge, MA 02139, USA

^4^ Department of Aeronautics and Astronautics, Massachusetts Institute of Technology, 77 Massachusetts. Avenue., Cambridge, MA 02139, USA

^5^ JJ Scientific, Mazowieckie, 02-792 Warsaw, Poland

^6^ Faculty of Environmental Engineering, Wroclaw University of Science and Technology, 50-370 Wroclaw, Poland

^*^ Correspondence: mihkel.pajusalu@ut.ee

# S1. Limits of active flight for animals on Earth

| Higher Clade of Animal Kingdom | Order | Maximum Highest Altitude Observed (km) / source | | Longest Continuous Travel Distance (km)^5^ / source | | Maximal Speed (km/h) / source | | Approximated atmospheric density (kg/m^3^) |
| --- | --- | --- | --- | --- | --- | --- | --- | --- |
| Insects (*Insecta*) | Mayflies (*Ephemenoptera*) | 5.0 (*Ephemera vulgata*) |  | (351)^1^ (*Ephemera vulgata*) | [1] | (15)^1^ (*Ephemera vulgata*) | [1] | 0.736 |
|  | Dragonflies and Damselflies (*Odonata*) | 2.5 (*Pantala flavescens*); 3.7 (*Aeshna* sp.) | [2,3] | 3500^4^ (*Pantala flavescens*) | [2,4] | 54 (98) (*Austrophlebia costalis*) | [5,6] | 0.969 |
|  | Moths and Butterflies (*Lepidoptera*) | 1.2 (*Autographa gamma*); 1.0 (*Ascia monusle*); 5.2 (*Vanessa cardui*); 5.5 (*Aglais cashmerensis*) | [7] | 700 (*Autographa gamma*); 400 (*Helicoverpa zea*); 2400 (4800) (*Danaus plexippus*); | [7–10] | 48 (96) (*Autographa gamma*); 28 (*Helicoverpa zea*); 113 (*Agrotis ipsilon*) | [8–11] | 1.096  1.117  0.713  0.690 |
|  | Ladybirds (*Coleoptera*) | 1.1 (*Coccinella septempunctata*) | [12] | 119 (*Coccinella septempunctata*) | [12] | 60 (*Coccinella septempunctata*) | [12] | 1.099 |
|  | Stoneflies (*Plecoptera*) | 5.0 |  |  |  |  |  | 0.736 |
|  | Locusts (*Locusta migratoria danica*) | 4.5; 2.1 (*Locusta migratoria danica*) | [3,7] | 130 (*Locusta migratoria danica*); 150 (*Schistocerca gregaria*) | [13] | 20 (*Locusta migratoria danica*); 33 (*Schistocerca gregaria*) | [10] | 0.777  0.996 |
|  | True bugs (*Hemiptera*) | 5.0 |  | 400^6^ (*Alcaeorrhynchus grandis*) | [14] |  |  | 0.736 |
|  | Caddisflies (*Trichoptera*) | 5.0 |  |  |  |  |  | 0.736 |
|  | Flies (*Diptera*) | 6.0 |  | 100 (*Hybomitra* sp.); 50 (*Aedes* sp.); 48 (*Drosophila* sp.) | [6] | (145)^2^ (*Hybomitra hinei wright*) | [15,16] | 0.660 |
|  | Hymenopterans (*Hymenoptera*) | 9.0^3^ (*Bombus impetuosus*) | [17,18] | 46 (*Apis mellifera*) | [6] |  |  | 0.466 |
| Birds | *Accipitriformes* | 11.3 (*Gyps rueppellii*); 7.3 (*Gypaetus barbatus*) | [19] | 150 (*Gyps rueppellii*) | [20] | 35 (*Gyps rueppellii*); 129 (*Aquila chrysaetos*) | [20,21] | 0.356  0.561 |
|  | *Procellariiformes* |  |  | 1000 (*Thalassarche chrysostoma*); 16000^7^ (*Diomedea exulans*) | [22,23] | 127 (*Thalassarche chrysostoma*) | [22] |  |
|  | *Apodiformes* | 3.0 (*Apus apus*) | [24] | 5000 (*Tachymarptis melba*); 10000^8^ (*Apus apus*) | [25,26] | 112 (*Apus apus*) |  | 0.909 |
|  | *Gruiformes* | 10.0 (*Grus grus*) | [27] | 480 (800) (*Grus grus*) | [28,29] | 56 (*Grus grus*) | [28,29] | 0.413 |
|  | *Anseriformes* | 8.8 (*Anser indicus*); 8.2 (*Cygnus cygnus*) | [27,30–32] | 1600 (*Anser indicus*) | [30] | 80 (160) (*Anser indicus*) | [30] | 0.478  0.513 |
|  | *Passeriformes* | 8.0 (*Pyrrhocorax graculus*) | [30] |  |  |  |  | 0.522 |
|  | *Cathartiformes* | 6.5 (*Vultur gryphus*) | [33] |  |  |  |  | 0.613 |
|  | Common cuckoo (*Cuculus canorus*) | 6.4 |  |  |  | 48 |  | 0.631 |
|  | Mallard (*Anas platyrhynchos*) | 6.4 | [30,31] |  |  |  |  | 0.631 |
|  | *Charadriiformes* | 6.0 (*Limosa lapponica*) | [30,34] |  |  |  |  | 0.660 |
|  | *Falconiformes* |  |  |  |  | 110 (*Falco peregrinus*); 145 (*Falco rusticolus*); |  |  |
|  | *Ciconiiformes* | 4.8 (*Ciconia Ciconia*) | [34] |  |  |  |  | 0.756 |
| Mammals | *Chiroptera* | 3.1 (*Tadarida brasiliensis*); 2.5 (*Lasiurus cinereus*); 1.5 (*Pteropus poliocephalus*) | [35–39] |  |  | 25 (*Eptesicus fuscus*); 160 (*Tadarida brasiliensis*) | [40,41] | 0.912  0.957  1.058 |
| Reptiles | *Pterosauria* | (4.5) (*Quetzalcoatlus northropi*) | [42] |  |  | (128) (*Quetzalcoatlus northropi*) | [42] | 0.786 |
| Fish | *Characiformes* | 0.001 (*Gasteropelecidae*) | [43] | 0.006 (*Gasteropelecidae*) | [43] |  |  | 1.225 |

**Table S1**. Animal Limits of Active Flight on Earth. Values in brackets ( ) are less reliable, or with a tail wind. ^1^ Theoretical maximal speed extrapolated based on modeling. ^2^ Interpolation of maximal speed based on slow-motion cinematography [16] ^3^ Extrapolated from laboratory experiments. ^4^ The maximum long-distance travel of *Pantala flavescens* is estimated to be 18000 km [44], however the likely documented continuous travel distance (e.g. from India to Somalia) is 3500 km [2]. ^5^ Note that it is very difficult to estimate the maximal travel distance of animals, especially insects. Some authors suggest that the maximum range of any insect, that is unable to feed while flying, is 1000 km [6]. The value of 1000 km proposed by [6] is in contrast to several migration studies that suggest that e.g. Monarch butterfly (*Danaus plexippus*) or other butterflies have ranges of many thousands of kilometers [7], although it is unclear how many times they stop during the migration. It is known that the Monarch butterfly can fly continuously for 117 hours without feeding [7]. ^6^ Minimal range, likely much larger than 400 km. ^7^ If dynamic soaring is involved in sustained non-flapping flight. ^8^ Likely an unlimited distance. It is likely that common swifts (*Apus apus*) have no limit to flight distance. It is very likely that they can perform most of their physiological functions while inflight, even sleeping like in the case of Great Frigatebird [24].

# Supplementary Dataset S1:

Supplementary Dataset S1 contains the following video files recorded from the stereo camera no.1: 

1) flies_2_N2_gstream1.mp4 - video showing the experiment with flushing with N2 (first repetition).

2) flies_2_N2_2_gstream1_1.mp4 - video showing the experiment with flushing with N2 (second repetition).

3) flies_2_N2_3_gstream1_1.mp4 - video showing the experiment with flushing with N2 (third repetition).

4) flies_1__He_gstream1_1.mp4 - video showing the experiment with flushing with He (first repetition).

5) flies_2_He_2_gstream1_1.mp4 - video showing the experiment with flushing with He (second repetition).

6) flies_2_He_3_gstream1_1.mp4 - video showing the experiment with flushing with He (third repetition).

All six original video files are available for download from Zenodo at <https://zenodo.org/records/11060392>.

# Supplementary References

1. Watson, T. How far could a mayfly travel in a day? *J. Interdiscip. Sci. Top.* **2018**, *7*, 4–5.

2. Anderson, R.C. Do dragonflies migrate across the western Indian Ocean? *J. Trop. Ecol.* **2009**, *25*, 347–358.

3. Mani, M.S. *Ecology and biogeography of high altitude insects*; Springer Science & Business Media, 2013; Vol. 4; ISBN 9401713391.

4. Russell, R.W.; May, M.L.; Soltesz, K.L.; Fitzpatrick, J.W. Massive swarm migrations of dragonflies (Odonata) in eastern North America. *Am. Midl. Nat.* **1998**, *140*, 325–342.

5. Tillyard, R.J. *The biology of dragonflies:(Odonata or Paraneuroptera)*; CUP Archive, 1917;

6. Hocking, B. The intrinsic range and speed of flight of insects. *Trans. R. Entomol. Soc. London* **1953**, *104*, 223–345.

7. Fowler, M.S.; Lagrone, A.H. Comparison of insects’ flight characteristics with observed characteristics of radar dot angels. *J. Appl. Meteorol.* **1969**, *8*, 122–127.

8. Chapman, J.W.; Nesbit, R.L.; Burgin, L.E.; Reynolds, D.R.; Smith, A.D.; Middleton, D.R.; Hill, J.K. Flight orientation behaviors promote optimal migration trajectories in high-flying insects. *Science (80-. ).* **2010**, *327*, 682–685.

9. Fang, J. Moths catch the wind to speed migration. *Nature* **2010**, doi:10.1038/news.2010.54.

10. Dean, T.J. Chapter 1: Fastest Flyer. In *Book of Insect Records*; School of Physics, University of New South Wales at the Australian Defence Force Academy, 2003.

11. Hardin, B. Corn pest logs thousands of frequent flyer miles. *Agric. Res.* **1992**, *40*, 20–24.

12. Jeffries, D.L.; Chapman, J.; Roy, H.E.; Humphries, S.; Harrington, R.; Brown, P.M.J.; Handley, L.-J.L. Characteristics and drivers of high-altitude ladybird flight: insights from vertical-looking entomological radar. *PLoS One* **2013**, *8*.

13. Draper, J. The direction of desert locust migration. *J. Anim. Ecol.* **1980**, 959–974.

14. Alves, R.J. V; Costa, L.A.A.; Soares, A.; Silva, N.G.; Pinto, Â.P. Open ocean nocturnal insect migration in the Brazilian South Atlantic with comments on flight endurance. *PeerJ* **2019**, *7*, e7583, doi:10.7717/peerj.7583.

15. Wilkerson, R.C.; Butler, J.F. The Immelmann Turn, a pursuit maneuver used by hovering male Hybomitra hinei wrighti (Diptera: Tabanidae). *Ann. Entomol. Soc. Am.* **1984**, *77*, 293–295.

16. Byrd, J.H. Chapter 1: Fastest Flyer. In *Book of Insect Records*; Department of Entomology & Nematology, University of Florida: Gainesville, 1994.

17. Dillon, M.E.; Dudley, R. Surpassing Mt. Everest: extreme flight performance of alpine bumble-bees. *Biol. Lett.* **2014**, *10*, 20130922.

18. Williams, P.H.; Ito, M.; Matsumura, T.; Kudo, I. The bumblebees of the Nepal Himalaya (Hymenoptera: Apidae). *Insecta matsumurana. New Ser. J. Fac. Agric. Hokkaido Univ. Ser. Entomol.* **2010**, *66*, 115–151.

19. Laybourne, R.C. Collision between a vulture and an aircraft at an altitude of 37,000 feet. *Wilson Bull.* **1974**, *86*, 461–462.

20. The Animal Files: Rüppell’s Vulture Available online: https://tinyurl.com/y7n4le7k.

21. Kochert, M.N.; Steenhof, K.; McIntyre, C.L.; Craig, E.H.; Poole, A.; Gill, F. The birds of North America, No. 684. *Acad. Nat. Sci. Philadelphia, Phialdelphia, PA, Am. Ornithol. Union, Washington, DC* **2002**.

22. Catry, P.; Phillips, R.A.; Croxall, J.P. Sustained Fast Travel by a Gray-Headed Albatross (Thalassarche Chrysostoma) Riding an Antarctic Storm. *Auk* **2004**, *121*, 1208–1213, doi:10.1093/auk/121.4.1208.

23. Sachs, G.; Traugott, J.; Nesterova, A.P.; Bonadonna, F. Experimental verification of dynamic soaring in albatrosses. *J. Exp. Biol.* **2013**, *216*, 4222 LP – 4232, doi:10.1242/jeb.085209.

24. Dokter, A.M.; Åkesson, S.; Beekhuis, H.; Bouten, W.; Buurma, L.; van Gasteren, H.; Holleman, I. Twilight ascents by common swifts, Apus apus, at dawn and dusk: acquisition of orientation cues? *Anim. Behav.* **2013**, *85*, 545–552, doi:https://doi.org/10.1016/j.anbehav.2012.12.006.

25. Liechti, F.; Witvliet, W.; Weber, R.; Bächler, E. First evidence of a 200-day non-stop flight in a bird. *Nat. Commun.* **2013**, *4*, 2554, doi:10.1038/ncomms3554.

26. Hedenström, A.; Norevik, G.; Warfvinge, K.; Andersson, A.; Bäckman, J.; Åkesson, S. Annual 10-month aerial life phase in the common swift Apus apus. *Curr. Biol.* **2016**, *26*, 3066–3070.

27. Carwardine, M. *Natural History Museum Book of Animal Records*; New York: Firefly Books Ltd, c2013, 2018;

28. Sandhill Crane Facts Available online: https://tinyurl.com/y86edmmb.

29. Top Ten Facts About Crane Migration Available online: https://tinyurl.com/yb9gpayo.

30. Whiteman, L. The High Life. *Audubon* **2000**, *102*, 104–108.

31. Lincoln, F.C.; Anastasi, P.A.; Hines, B.; Peterson, S.R.; Zimmerman, J.L. *Migration of Birds*; Circular (U.S. Fish and Wildlife Service); U.S. Government Printing Office, 1999; ISBN 9780160617010.

32. Harrison, J. The highs and lows of bird flight. *Elife* **2019**, *8*, e50626.

33. Gargiulo, C.N. Distribución y situación actual del cóndor andino (Vultur gryphus) en las sierras centrales de Argentina 2012.

34. Elphick, J. *Atlas of bird migration*; Firefly Books, 2007; ISBN 1554072484.

35. McCracken, G.F. Bats aloft: a study of high-altitude feeding. *Bats* **1996**, *14*, 7–10.

36. Voigt, C.C.; Currie, S.E.; Fritze, M.; Roeleke, M.; Lindecke, O. Conservation strategies for bats flying at high altitudes. *Bioscience* **2018**, *68*, 427–435.

37. Williams, T.C.; Ireland, L.C.; Williams, J.M. High altitude flights of the free-tailed bat, Tadarida brasiliensis, observed with radar. *J. Mammal.* **1973**, *54*, 807–821.

38. Peurach, S.C. High-altitude collision between an airplane and a hoary bat, Lasiurus cinereus. *Bat Res. News* **2003**.

39. Parsons, J.G.; Blair, D.; Luly, J.; Robson, S.K.A. Flying-fox (Megachiroptera: Pteropodidae) flight altitudes determined via an unusual sampling method: aircraft strikes in Australia. *Acta Chiropterologica* **2008**, *10*, 377–379.

40. Hayward, B.; Davis, R. Flight Speeds in Western Bats. *J. Mammal.* **1964**, *45*, 236–242, doi:10.2307/1376986.

41. McCracken, G.F.; Safi, K.; Kunz, T.H.; Dechmann, D.K.N.; Swartz, S.M.; Wikelski, M. Airplane tracking documents the fastest flight speeds recorded for bats. *R. Soc. open Sci.* **2016**, *3*, 160398.

42. Witton, M.P.; Habib, M.B. On the size and flight diversity of giant pterosaurs, the use of birds as pterosaur analogues and comments on pterosaur flightlessness. *PLoS One* **2010**, *5*.

43. Frey, H. *Illustrated dictionary of tropical fishes*; TFH Publications: New Jersey, 1961;

44. Troast, D.; Suhling, F.; Jinguji, H.; Sahlén, G.; Ware, J. A global population genetic study of Pantala flavescens. *PLoS One* **2016**, *11*.
